# The Complex Role of STAT3 in Viral Infections

**DOI:** 10.1155/2015/272359

**Published:** 2015-06-25

**Authors:** Suresh V. Kuchipudi

**Affiliations:** School of Veterinary Medicine and Science, University of Nottingham, Sutton Bonington LE12 5RD, UK

## Abstract

Signal transducer and activators of transcription-3 (STAT3) regulates diverse biological functions including cell growth, differentiation, and apoptosis. In addition, STAT3 plays a key role in regulating host immune and inflammatory responses and in the pathogenesis of many cancers. Several studies reported differential regulation of STAT3 in a range of viral infections. Interestingly, STAT3 appears to direct seemingly contradictory responses and both pro- and antiviral roles of STAT3 have been described. This review summarized the currently known functions of STAT3 in the regulation of viral replication and pathogenesis of viral infections. Some of the key unanswered questions and the gap in our current understanding of the role of STAT3 in viral pathogenesis are discussed.

## 1. Introduction

Signal transducers and activators of transcription (STATs) are a family of transcription factors that play crucial roles in regulating a number of diverse biological functions including cell proliferation, differentiation, apoptosis, inflammatory response, immunity, and angiogenesis [1]. There are seven STAT proteins (STATs 1, 2, 3, 4, 5a, 5b, and 6), which are activated by the signals from cytokine and growth factor receptors in the plasma membrane and regulate gene transcription [2]. A unique feature of STAT proteins is their dual roles, which include signal transduction through the cytoplasm and functioning as transcription factors in the nucleus [3–5].

STATs were first discovered through their capacity to mediate signalling from interferon (IFN) and interleukin-6 (IL-6) receptors following binding of cytokines [3, 4, 6–8]. Each STAT family protein responds to a defined set of cytokines (Table 1), and certain cytokines can activate more than one STAT protein [9]. Cytokine receptors on plasma membrane do not usually possess intrinsic tyrosine kinase activity and their engagement activates receptor-associated tyrosine kinases, prominent of which are Janus kinase (JAK) family kinases (JAK1, JAK2, JAK3, and TYK2) [3, 4, 8–10].

STAT proteins are activated by phosphorylation of specific tyrosine residues, following which they form stable homodimers or heterodimers with other STAT proteins through reciprocal phosphotyrosine-SRC homology 2 (SH2) domain interactions [9]. STAT dimers then translocate to nucleus where they regulate the transcription of a set of specific genes. A number of the downstream target genes of STATs encode cytokines and growth factors, which in turn mediate autocrine and paracrine STAT activation [9].

## 2. Noncanonical STAT Activation

In addition to the canonical model of JAK/STAT signalling, STATs also form dimers in the absence of the activating tyrosine phosphorylation [11]. In the noncanonical model, unphosphorylated STATs are consistently found as a result of constant nuclear import and export. These unphosphorylated nuclear STAT molecules might also contribute to gene regulation [12].

## 3. STAT3

Of all the STAT family proteins, STAT3 is unique as it is known to direct seemingly contradictory responses [13] and is essential for early embryonic development in mice [14]. STAT3 regulates cell-cycle progression and apoptosis, plays a key role in oncogenesis [15], and is aberrantly expressed in cancer cells [16]. STAT3 function has been extensively studied in cell culture systems. STAT3 is known to regulate a number of distinct responses in different cells, including induction of an acute-phase response in hepatoma cells, stimulation of proliferation in B lymphocytes, activation of terminal differentiation and growth arrest in monocytes [4], and maintenance of the pluripotency of embryonic stem cells [13, 17–20]. The seemingly contradictory responses of STAT3 could be explained in part by the activation of distinct sets of target gene by STAT3 in different cells [21].

## 4. STAT3 Domain Structure 

**Figure 1:**
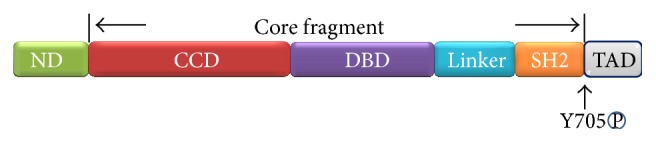
Structure of STAT3. The six domains of STAT3 are N-terminal domain (ND), coiled-coil domain (CCD), DNA-binding domain (DBD), linker domain, SH2 domain, and transcriptional activation domain (TAD). Between SH2 and TAD there is a tail segment that contains the phosphorylation site Y705.

The structure of STAT3 is similar to the other STAT family members comprising six structural regions, namely, N-terminal domain (ND), coiled-coil domain (CCD), DNA-binding domain (DBD), linker domain, SH2 domain, and a C-terminal transcriptional activation domain (TAD) (Figure 1). The core fragment of STAT3 comprising CCD, DBD, linker, and SH2 domains is monomeric and the dimer interface observed in the unphosphorylated STAT1 core fragment structure is absent in the STAT3 structure [22].

## 5. STAT3 Activation

STAT3 is activated by phosphorylation at tyrosine 705 in the C-terminal domain [2] and more than 40 different polypeptide ligands are known to cause STAT3 phosphorylation [16]. STAT3 was initially described as a DNA-binding factor that is capable of selectively interacting with an enhancer element in the promoter region of acute-phase genes in interleukin-6 (IL-6) stimulated hepatocytes [7]. Subsequently, it became evident that STAT3 can be activated by the entire IL-6 family and other cytokines including leukemia inhibitory factor (LIF), cardiotrophin-1, ciliary neurotrophic factor (CNTF), IL-5, IL-9, IL-10, IL-11, IL-12, IL-21, IL-22, IL-27, IFN-*γ*, TNF-*α*, LIGHT, a member of the TNF superfamily, monocyte chemotactic protein-1 (MCP-1), macrophage inflammatory protein-1*α* (MIP-1*α*), CCL-5/RANTES, stem cell factor (SCF), and oncostatin M (OSM) [23–38].

Various growth factor receptors also activate STAT3, which include epidermal growth factor receptors (EGFRs), hepatocyte growth factor receptors (HGFRs), fibroblast growth factor receptors (FGFRs), platelet-derived growth factor receptors (PDGFRs), insulin-like growth factor receptors (IGFRs), and vascular endothelial growth factor receptors (VEGFRs) [39]. In addition, many carcinogenic agents such as nicotine in cigarette smoke, diesel exhaust particles, bacterial lipopolysaccharide (LPS), environmental stress including ultraviolet light, osmotic shock, heat shock, and oxidative stress, Ca^2+^/calmodulin-dependent protein kinase II*γ* (CaMKII*γ*), bile acids, leptin and low pH, black soy peptides, diazoxide, isoliquiritigenin, and olanzapine have been found to activate STAT3 (reviewed by Siveen et al., 2014 [16]).

## 6. STAT3 Signal Transduction

Growth factor or cytokine receptor-ligand interaction results in dimerization of gp130, a signal transducer protein in cytoplasm [40]. This is followed by phosphorylation of JAK family of tyrosine kinases especially JAK1 which in turn mediates STAT3 phosphorylation [21]. STAT3 has two important phosphorylation sites at Tyr705 and Ser727. Ser727 phosphorylation has been considered to be a secondary event after Tyr705 phosphorylation. However, recent evidence suggests that Ser727 phosphorylation on STAT3 is not necessarily a secondary event after Tyr705 phosphorylation but has a role in the regulation of cell survival activity and nuclear translocation of STAT3 in melanocytic cells [41]. Further, STAT3 phosphorylation at serine 727 in the C-terminal transactivation domain promotes maximal transcriptional activation of a subset of target genes [42]. Furthermore, Ser727 phosphorylation of STAT3 appears to be important in tumorigenicity. For example, in glioma, reduced STAT3 Ser727 phosphorylation enhances tumorigenicity which may be regulated in part by CK2-PP2A pathway [43].

**Figure 2:**
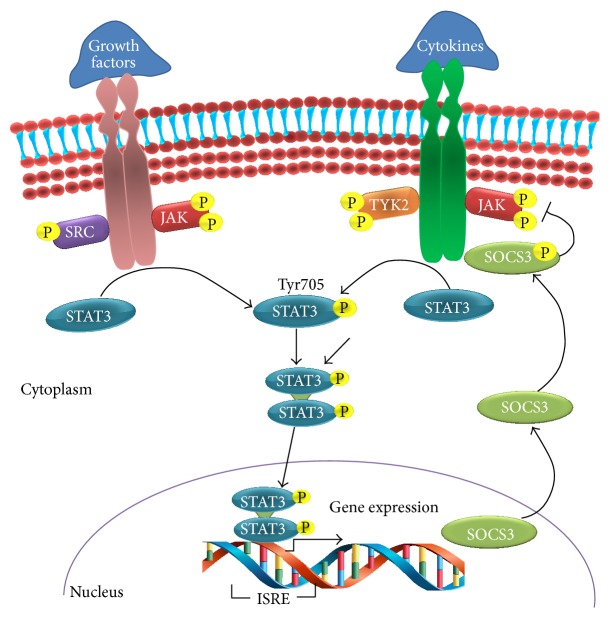
STAT3 signal transduction pathway. In response to cytokines and growth factors, STAT3 is phosphorylated by receptor-associated kinases and then forms homo- or heterodimers. STAT3 dimers then translocate to the nucleus where they act as a transcription activator and mediate the expression of a variety of genes. STAT3 activates the transcription of suppressors of cytokine signalling 3 (SOCS3) which act as classical feedback inhibitor of STAT3 activation.

The phosphorylation of STAT3 promotes homodimerization, wherein the SH2 domain of each STAT3 monomer interacts with the Y705 residue on another monomeric STAT3 [2]. STAT3 homodimers translocate into the nucleus which is mediated by importin *α*5/NPI-1 [44] and bind to specific DNA response elements such as the IFN-stimulated response element (ISRE) in the promoter regions of responsive target genes and regulate their transcription [45–47] (Figure 2).

STAT3 dimers recognize an 8- to 10-base pair inverted repeat DNA element with a consensus sequence of 5-TT(N)AA-3 [15]. Further, nonphosphorylated STAT3 monomers are also capable of dimerization and induction of transcription through binding to NFkappaB [48].

## 7. Regulation of STAT3 Activation

Activation of JAK/STAT pathway begins with the binding of extracellular signalling proteins (ESPs) to specific receptors associated with JAKs. Activated JAKs then activate STAT proteins by phosphorylation which otherwise remain latent in the cytoplasmic [49, 50]. A range of endogenous protein regulators tightly control the receptor-induced STAT3 activation [4, 51–53]. Several protein tyrosine phosphatases (PTPs) including SHP-2, PTP1B, PTP*ε*C, TC45, and SHP-1 have been implicated in the termination of STAT3 signaling. Because only STAT3 dimers bind to DNA, the nuclear PTP TC45 may be important in the termination of STAT3-mediated transcriptional activation [51, 53].

Protein inhibitors of activated STAT (PIAS) family members are involved in blocking the DNA-binding ability of STAT proteins thereby inhibiting their function as transcription factors. PIAS family of proteins shares a highly conserved domain structure comprising the N terminus SAP domain that can bind to AT-rich DNA sequences and a Pro-Ile-Asn-Ile-The (PINIT) motif, which is involved in the nuclear retention of PIAS proteins. PIAS3 has been shown to specifically bind to STAT3 and block its DNA-binding activity and transcriptional activation [2, 52, 54].

The family of suppressors of cytokine signalling (SOCS) acts as classical feedback inhibitors of the JAK/STAT pathway. STAT3 activates the transcription of SOCS3 which can block STAT3 signalling either by direct binding and inhibition of JAKs, by competing with STAT3 for pY-binding sites on activated receptor chains, or by binding signalling proteins and targeting them for proteasomal degradation [55, 56] (Figure 2).

## 8. Role of STAT3 in Regulating Cytokine Response

STAT3 is a signaling mediator of IL-6 and IL-10 family members and other cytokines such as leptin and G-CSF [13, 57]. N-terminal domain of STAT3 negatively regulates type 1 interferon (IFN) response in mice, which is independent of its function as a transcriptional factor [58]. While STAT1 and STAT2 mediate antiviral and inflammatory effects of type I IFNs, STAT3 has been shown to negatively regulate the inflammatory properties of type I IFNs possibly through suppression of STAT1 function [59].

STAT3 activation results in either activation or suppression of inflammatory response depending on the physiological status of the cells. STAT3 is found to be constitutively activated in cancer cells and the persistent activation of STAT3 in cancer cells mediates tumour-promoting inflammation [9]. STAT3 also promotes a potent anti-inflammatory response (AIR); for example,* Toxoplasma gondii* exploits host STAT3 to prevent LPS-triggered proinflammatory cytokine production in infected mouse macrophages [60]. IL-10 regulates both acute and chronic inflammation [61] and STAT3 is essential for all known aspects of the IL-10-regulated anti-inflammatory effect both* in vivo* and* in vitro* [62–64]. While it is not clear how the pro- and anti-inflammatory functions of STAT3 are regulated, possible factors could be the physiological status of the cells and the length of STAT3 activation during a response.

## 9. Regulation of STATs by Viruses

A number of viruses are known to regulate STAT family members including STAT1, STAT2, and STAT3. Epstein-Barr virus infection results in transcriptional activation of STAT1 through latent membrane protein 1 (LMP1) [65]. Dengue virus subverts the IFN response in infected human cells by downregulating STAT2 expression [66].

## 10. STAT3 Regulation by Viruses

Several studies described the involvement of STAT3 in the replication and pathogenesis of viruses in humans and animals. Notably, both pro- and antiviral functions of STAT3 have been documented and its precise role in the pathogenesis of viral infections is not yet fully established. A number of DNA and RNA viruses are known to regulate STAT3, which is summarized in Table 2.

**Figure 3:**
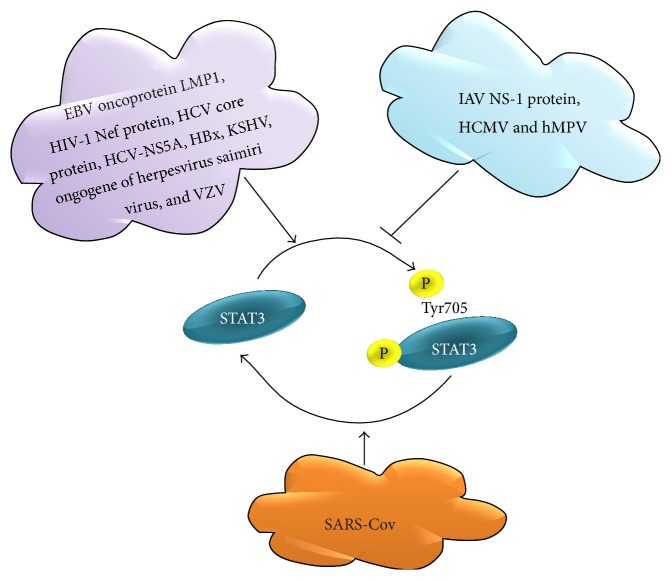
STAT3 regulation in viral infections. Epstein-Barr virus (EBV) oncoprotein latent membrane protein 1 (LMP1), human immunodeficiency virus type 1 (HIV-1) Nef protein, hepatitis C virus (HCV) core protein, HCV nonstructural protein 5A (NS5A), hepatitis B virus X protein (HBx), Kaposi's sarcoma-associated herpesvirus (KSHV), saimiri transforming protein (STP) oncogene of* Herpesvirus saimiri* subgroup A strain 11 (STP-A11), and Varicella-zoster virus (VZV) activate STAT3 phosphorylation. Influenza A virus (IAV) nonstructural protein 1 (NS-1) human metapneumovirus (hMPV) and human cytomegalovirus (HCMV) inhibit STAT3 phosphorylation. Severe acute respiratory syndrome coronavirus (SARS-CoV) infection results in STAT3 dephosphorylation.

STAT3 is either positively or negatively regulated in a range of viral infections depending on the type of virus involved.  Figure 3 provides a summary of positive or negative regulation of STAT3 by viruses. A number of viral proteins interact with STAT3 resulting in the phosphorylation activation. Epstein-Barr virus (EBV) oncoprotein latent membrane protein 1 (LMP1) [67], human immunodeficiency virus type 1 (HIV-1) Nef protein [68], hepatitis C virus (HCV) core protein [69], HCV nonstructural protein 5A (NS5A) [70], mitochondrially associated hepatitis B virus X protein (HBx) [71], Kaposi's sarcoma-associated herpesvirus (KSHV) [72], saimiri transforming protein (STP) oncogene of* Herpesvirus saimiri* subgroup A strain 11 (STP-A11) [73], and Varicella-zoster virus (VZV) [74] promote STAT3 phosphorylation in infected cells.

In contrast, influenza A virus (IAV) nonstructural protein 1 (NS-1) [75] and human metapneumovirus [76] impede STAT3 phosphorylation in infected cells. However, human cytomegalovirus (HCMV) infection inhibits STAT3 phosphorylation but rapidly promotes nuclear localization of unphosphorylated STAT3 to the nucleus and disrupts IL-6-induced gene expression [77]. Severe acute respiratory syndrome coronavirus (SARS-CoV) infection of Vero E6 cells results in STAT3 dephosphorylation at Tyr705 [78]. In addition HCV promotes STAT3 ubiquitination and degradation via the proteasome [79].

## 11. Role of STAT3 in Mediating Host Immune Responses to Viruses

STAT3 plays an important role in adaptive immune response, in particular in the regulation of T lymphocyte function. STAT3 mediates IL-6-dependent T cell proliferation by preventing apoptosis [80]. Further, STAT3 regulates proliferation, survival, and differentiation of CD4^+^ [81] and CD8^+^ T cells [82]. Furthermore, STAT3 is essential in upregulating CD8^+^ T cell-mediated responses to viruses. For example, STAT3 plays an important role in the activation of CD8^+^ T cells effective response during herpes simplex virus (HSV-1) infection [82].

It is evident that viruses either promote or disrupt STAT3-mediated gene transcription and host immune responses against viruses. Further, activation or inhibition of STAT3-mediated signalling appears to be dependent on virus and host cell type involved. Inhibition of STAT3 activation could be a strategy of viruses to subvert host immune responses. On the other hand STAT3-mediated gene transcription of proviral factors could be essential for certain other viruses. It is possible that the timing of STAT3 regulation during virus infection may be critical and hence require further in depth studies to profile STAT3 regulation during different stages of viral infection.

## 12. Role of STAT3 in Virus Replication

The role of STAT3 in virus replication appears to be complex, as it appears to function as a proviral factor in some viral infections and antiviral factor in others. STAT3 cooperatively interacts with hepatocyte nuclear factor 3 (HNF-3) and activates hepatitis B virus (HBV) gene expression [83]. There is a STAT3 binding site within the core domain of hepatitis B virus (HBV) enhancer 1. IL-6 and epidermal growth factor stimulates the interaction of HBV enhancer 1 DNA-STAT3 protein resulting in overall stimulation of HBV enhancer 1 function and viral gene expression [83].

HCV constitutively activates STAT3 in liver cells which plays an important role in HCV RNA replication [84]. HCV replicon-expressing cells showed constitutive activation of STAT3 which is mediated by oxidative stress and influenced by the activation of cellular kinases, including p38 mitogen-activated protein kinase, JNK, JAK-2, and Src [84].

HCMV primarily utilizes unphosphorylated STAT3 to promote, either directly or indirectly, the initiation of HCMV DNA replication [77]. HCMV infection disrupts IL-6 induced phosphorylation of STAT3 and expression of a subset of IL-6-induced STAT3-regulated genes including SOCS3. HCMV 72-kDa immediate-early 1 (IE1) protein associates with STAT3 and rapidly promotes nuclear localization of STAT3 in the absence of robust phosphorylation at Y705 and inhibition of STAT3 nuclear localization or STAT3 expression during infection results in diminished HCMV genome replication [77].

STAT3 activation was shown to be critical for replication of VZV that causes varicella (chickenpox) during primary infection and zoster (shingles) upon reactivation [74]. VZV triggers STAT3 phosphorylation in cells infected* in vitro* and also in human skin xenografts in SCID mice* in vivo*. STAT3 activation induces the antiapoptotic protein survivin and both STAT3 and surviving are essential for VZV replication as inhibition of STAT3 phosphorylation and survivin results in restricted VZV replication [74].

Mumps virus V protein functions as a ubiquitin ligase that targets STAT3 for degradation and STAT3 evasion has been proposed to be beneficial to the replication of Paramyxoviruses [85]. Further, mumps virus V protein prevents responses to interleukin-6 and v-Src signals and can induce apoptosis in STAT3-dependent multiple myeloma cells and transformed murine fibroblasts [85, 86]. In a similar fashion, measles virus also interferes with STAT3 activation and it was proposed that this could provide several general or tissue-specific replication advantages to the virus [86].

Influenza A virus (IAV) NS1 protein interferes with IFN production [87] which correlates to reduced phosphorylation of STAT3. Transfection of NS1 from a highly pathogenic avian influenza (HPAI) H5N1 virus in human lung epithelial A549 cells resulted in a notable reduction in IFN-inducible STAT3 phosphorylation [75].

Viruses such as HBV, HCV, and HCMV appear to exploit host genes transcriptionally regulated by STAT3 for their gene replication which could be independent of the regulatory effects of STAT3 on type 1 IFN response. Whereas STAT3 inhibition appears to be a strategy of viruses such as Paramyxo- and Orthomyxoviruses to evade host innate and adaptive immune responses, it is plausible that inhibition of STAT3 signalling could provide a much broader spectrum of cytokine and growth factor suppression to allow replication and spread of these viruses* in vivo.*

## 13. Role of STAT3 in the Pathogenesis of Viral Infections

STAT3 role in viral pathogenesis appears to be complex with reports suggesting both promotion of innate antiviral response and contribution to the detrimental effects of viral infection. STAT3 is constitutively phosphorylated in neoplastic cells [88] and many viruses exploit the oncogenic effects of phosphorylated STAT3 (pSTAT3). STAT3 plays a central role in the pathogenesis of oncogenic viruses such as the *γ*-herpesviruses, KSHV, EBV, and* Herpesvirus saimiri* [72, 73, 89].

STAT3 role has been well characterized in the pathogenesis of viral infections resulting in liver disease in humans. HCV infection in liver cells causes constitutive activation of STAT3, which plays a central role in chronic hepatitis and often results in liver cirrhosis and hepatocellular carcinoma [84]. HCV core protein directly interacts with and activates STAT3 through phosphorylation of the critical tyrosine and is responsible for the virus-induced transformation. Activation of STAT3 by the HCV core results in rapid proliferation and upregulation of Bcl-XL and cyclin-D1 and additional expression of STAT3 in HCV core-expressing cells results in anchorage-independent growth and tumorigenesis [90]. Cytokine stimulation of HBV gene expression represents an important regulatory scheme of direct relevance to the pathogenesis of liver disease associated with HBV infection [83]. HCMV infection of PHH and HepG2 cells results in activation of the IL-6-JAK-STAT3 pathway which results in the transformation of PHH cells and enhanced HepG2 tumorsphere formation raising the possibility that HCMV infection might be involved in the genesis of hepatocellular carcinoma [91].

STAT3 activation and upregulation of antiapoptotic protein survivin play an important role in the pathogenesis of lytic as well as tumorigenic herpesviruses [74]. STAT3 and survivin have been shown to play a major role in the malignant transformation of cells infected by *γ*-herpesviruses, such as KSHV [72]. STAT3 activation is essential for the skin infections caused by VZV which is necessary for viral transmission and persistence in the human population [74].

## 14. Protective Role of STAT3 in Viral Diseases

STAT3 is also known to promote host defense against virus infections and play a protective role in regulating virus mediated proinflammation. Gp130-STAT3 signalling plays an important role in the innate immune response in cardiac myocytes against coxsackievirus B3 infection [92]. Dysregulation of host proinflammatory response is a key contributing factor to the morbidity and mortality of virulent influenza virus infections such as the highly pathogenic avian influenza (HPAI) H5N1 viruses [93–96]. We recently showed that elevated proinflammatory response in chickens is a major pathogenicity factor of HPAI-H5N1 virus infection possibly mediated by inhibition of STAT3 phosphorylation [96]. HPAI-H5N1 virus infection results in STAT3 inhibition and elevated proinflammatory response in chicken cells. In contrast STAT3 inhibition was not found in HPAI-H5N1 virus infected duck cells which show a moderate proinflammatory response.

In summary, STAT3 plays a significant role in the complex interplay between viruses and their hosts and functions as either a pro- or antiviral factor depending on the virus and host cell type involved. While STAT3 role has been reasonably well characterized in the pathogenesis of oncogenic viruses and viruses causing liver pathology, its role in many other viral infections is less well understood. STAT3 regulates antiviral and proinflammatory responses, either through transcriptional regulation of other cellular factors or through pathways independent of its role as a transcription factor. The seemingly contradictory roles of STAT3 in viral infections raise a number of interesting questions: “what factors determine the switch between pro- and anti-inflammatory functions of STAT3?,” “how are viruses able to exploit STAT3 signalling for their gene replication?,” and “does STAT3 either negatively or positively regulate type 1 IFN response depending on the virus type involved?” Further in depth studies to dissect the role of STAT3 in viral infections could provide valuable insights into viral pathogenesis and development of novel antiviral therapies.* In vitro* studies using cell culture systems are a valuable tool to dissect the molecular basis of STAT3 role in innate response to viruses. However,* in vivo* studies are essential to elucidate STAT3 role in adaptive immune response to viruses. Complementary* in vitro* and* in vivo* experiments are therefore essential to better understand the seemingly contradictory role of STAT3 in viral infections.

## Figures and Tables

**Table 1:** Activators of STAT family proteins (adapted from Yu et al., 2009 [9]).

STAT protein	Key activators
STAT1	IFN*γ*, IFN*α*, and IFN*β*
STAT2	IFN*α* and IFN*β*
STAT3	IL-6, IL-10, IL-23, IL-21, IL-11, LIF, and OSM
STAT4	IL-12
STAT5A and STAT5B	IL-2, GM-CSF, IL-15, IL-7, IL-3, IL-5, growth hormones, and prolactin
STAT6	IL-4 and IL-13

**Table 2:** Viruses that are known to regulate STAT3 activation.

DNA viruses	RNA viruses
Epstein-Barr virus (EBV)	Human immunodeficiency virus type 1 (HIV-1)
Kaposi's sarcoma-associated herpesvirus (KSHV)	Hepatitis C virus (HCV)
*Herpesvirus saimiri* subgroup A strain 11 (STP-A11)	Influenza A virus (IAV)
Varicella-zoster virus (VZV)	Severe acute respiratory syndrome coronavirus (SARS-CoV)
Human cytomegalovirus (HCMV)	Mumps virus
Hepatitis B virus (HBV)	Measles virus
